# Supplementary material for: Genetic similarity between relatives provides evidence on the presence and history of assortative mating
Source: Nat Commun. 2024 Mar 26;15:2641. doi: 10.1038/s41467-024-46939-9 (PMC10966108; doi:10.1038/s41467-024-46939-9)
Supplement: Supplementary file 3 — Reporting Summary [file 41467_2024_46939_MOESM3_ESM.pdf]

nature portfolio

Corresponding author(s): Sunde, Hans Fredrik

Last updated by author(s): Feb 24, 2024

Reporting Summary

Nature Portfolio wishes to improve the reproducibility of the work that we publish. This form provides structure for consistency and transparency in reporting. For further information on Nature Portfolio policies, see our Editorial Policies and the Editorial Policy Checklist.

Statistics

For all statistical analyses, confirm that the following items are present in the figure legend, table legend, main text, or Methods section.

n/a	Confirmed	
☐	☒	The exact sample size (*n*) for each experimental group/condition, given as a discrete number and unit of measurement
☐	☒	A statement on whether measurements were taken from distinct samples or whether the same sample was measured repeatedly
☐	☒	The statistical test(s) used AND whether they are one- or two-sided<br>*Only common tests should be described solely by name; describe more complex techniques in the Methods section.*
☐	☒	A description of all covariates tested
☐	☒	A description of any assumptions or corrections, such as tests of normality and adjustment for multiple comparisons
☐	☒	A full description of the statistical parameters including central tendency (e.g. means) or other basic estimates (e.g. regression coefficient) AND variation (e.g. standard deviation) or associated estimates of uncertainty (e.g. confidence intervals)
☐	☒	For null hypothesis testing, the test statistic (e.g. *F*, *t*, *r*) with confidence intervals, effect sizes, degrees of freedom and *P* value noted<br>*Give P values as exact values whenever suitable.*
☒	☐	For Bayesian analysis, information on the choice of priors and Markov chain Monte Carlo settings
☒	☐	For hierarchical and complex designs, identification of the appropriate level for tests and full reporting of outcomes
☐	☒	Estimates of effect sizes (e.g. Cohen's *d*, Pearson's *r*), indicating how they were calculated

Our web collection on statistics for biologists contains articles on many of the points above.

Software and code

Policy information about availability of computer code

Data collection	No software was used for data collection (we used existing data)
Data analysis	R 4.0.3, OpenMx 2.20.6, KING (Kinship-based Inference for GWAS), LDPred v.1, Plink2<br><br>R script for running simulations are provided in Supplementary Software 1, and on https://osf.io/dgw4r/

For manuscripts utilizing custom algorithms or software that are central to the research but not yet described in published literature, software must be made available to editors and reviewers. We strongly encourage code deposition in a community repository (e.g. GitHub). See the Nature Portfolio guidelines for submitting code & software for further information.

Data

Policy information about availability of data

All manuscripts must include a data availability statement. This statement should provide the following information, where applicable:

- Accession codes, unique identifiers, or web links for publicly available datasets
- A description of any restrictions on data availability
- For clinical datasets or third party data, please ensure that the statement adheres to our policy

Data from the Norwegian Mother, Father and Child Cohort Study (MoBa) used in this study are managed by the national health register holders in Norway (Norwegian Institute of Public Health) and can be made available to researchers, provided approval from the Regional Committees for Medical and Health Research

Ethics (REC), compliance with the EU General Data Protection Regulation (GDPR) and approval from the data owners. The consent given by the participants does not open for storage of data on an individual level in repositories or journals. Researchers who want access to data sets for replication should apply through helsedata.no. Access to data sets requires approval from The Regional Committee for Medical and Health Research Ethics in Norway and an agreement with MoBa. The data in this study were accessed under ethics approval (project# 2017/2205, Regional Committees for Medical and Health Research Ethics, Southern and Eastern Norway).

Research involving human participants, their data, or biological material

Policy information about studies with human participants or human data. See also policy information about sex, gender (identity/presentation), and sexual orientation and race, ethnicity and racism.

Reporting on sex and gender	Neither the term "sex" or "gender" is used in the paper as the genetic consequences discussed herein should be the same regardless of sex. We do check whether mothers and fathers, identified by their relation to their offspring, have the same genetic variance, but otherwise do not consider sex.
Reporting on race, ethnicity, or other socially relevant groupings	The sample is homogenous and mostly of European ancestry, potentially constraining generalizability to similar populations. We do not consider their ethnicity, but non-europeans are excluded during genetic quality control (see methods, and Corfield 2023) because the discovery samples used for the polygenic indeces were based on europeans only. In addition, the polygenic indices were adjusted for 20 principal components to account for population stratification.
Population characteristics	See below
Recruitment	No participants were recruited for the current study
Ethics oversight	The current study was approved by The [Norwegian] Regional Committees for Medical and Health Research Ethics (2017/2205)

Note that full information on the approval of the study protocol must also be provided in the manuscript.

Field-specific reporting

Please select the one below that is the best fit for your research. If you are not sure, read the appropriate sections before making your selection.

☐ Life sciences ☒ Behavioural & social sciences ☐ Ecological, evolutionary & environmental sciences

For a reference copy of the document with all sections, see nature.com/documents/nr-reporting-summary-flat.pdf

Behavioural & social sciences study design

All studies must disclose on these points even when the disclosure is negative.

Study description	Population based pregnancy cohort. Observational quantitative data (incl. genomic data)
Research sample	The study is based on the Norwegian Mother, Father and Child Cohort study. The target population of the study is all women who give birth in Norway and their partners. There are no exclusion criteria. Studies of assortative mating need large samples of the general population, which this cohort study provided. The sample is largely representative for the Norwegian population, however, parents with high educational attainment were more likely to participate, which may reduce generalizability. The sample is homogenous and mostly of European ancestry, potentially constraining generalizability to similar populations.<br><br>The sample included all individuals who had been genotyped and passed quality control. This included 77,506 mothers (birth year: M = 1974.36, SD = 5.1), 53,274 fathers (birth year: M = 1972.27, SD = 5.6), and 71,525 children (49% female, birth year: M = 2005.31, SD = 1.94).
Sampling strategy	The current study was not conceived at the time of sampling, and therefore had no bearing on the sampling strategy used.<br><br>All hospitals and maternity units with more than 100 births annually, altogether 52 units, were to be included, and by January 2006, 50 units participated in the study. For practical reasons, the sampling frame comprised pregnant women who attended routine ultrasound examination. Together with appointments for ultrasound scanning in week 17–18 of pregnancy, the pregnant women received a postal invitation that included an informed consent form, the first questionnaire, an information brochure as well as consent form and questionnaire for the father.
Data collection	When she attended the ultrasound examination, the woman was asked whether she had consented to participate. If yes, the woman was referred to the laboratory for blood and urine samples and, if he consented, also a blood sample from the father. If a woman or a couple decided to participate in the study while attending the ultrasound examination, a consent form could be filled out there and then. Questionnaires were filled in using pen and paper, and then sent by mail to a central facility where they were registered, scanned, and verified. No questionnaire data was used in this study. Genetic data was obtained using the blood samples. Blood samples from the children were taken from the umbilical cord after birth.<br><br>The hypotheses were not conceived at the time of data collection - the researchers at that time were therefore blinded to the hypotheses. The authors of this paper were not blinded to the hypotheses.

Timing	Cohort recruited from July 1999 until December 2008. Data collection is still ongoing. Genotyped 2012-2021
Data exclusions	We used data for all individuals with valid and currently available genomic data.
Non-participation	The total participation rate for all invited pregnancies is 41% (112908/277702). Individuals who declined to participate were not asked to state a reason for this. Later, 146 families (0.1% of the recruited) withdrew their consent and had their data deleted (they did not have to state a reason for this).
Randomization	No randomization. Polygenic indices were adjusted for 20 principal components to account for population stratification.

Reporting for specific materials, systems and methods

We require information from authors about some types of materials, experimental systems and methods used in many studies. Here, indicate whether each material, system or method listed is relevant to your study. If you are not sure if a list item applies to your research, read the appropriate section before selecting a response.

Materials & experimental systems

n/a	Involved in the study
☒	☐ Antibodies
☒	☐ Eukaryotic cell lines
☒	☐ Palaeontology and archaeology
☒	☐ Animals and other organisms
☒	☐ Clinical data
☒	☐ Dual use research of concern
☒	☐ Plants

Methods

n/a	Involved in the study
☒	☐ ChIP-seq
☒	☐ Flow cytometry
☒	☐ MRI-based neuroimaging

Plants

Seed stocks	N/A
Novel plant genotypes	N/A
Authentication	N/A
